# Blending Internet-based and tele group treatment: Acceptability, effects, and mechanisms of change of cognitive behavioral treatment for depression

**DOI:** 10.1016/j.invent.2022.100551

**Published:** 2022-06-01

**Authors:** Raphael Schuster, Elena Fischer, Chiara Jansen, Nathalie Napravnik, Susanne Rockinger, Nadine Steger, Anton-Rupert Laireiter

**Affiliations:** a Department of Psychology, University of Salzburg, Austria; b PFH Göttingen, Germany; c Faculty of Psychology, University of Vienna, Austria

**Keywords:** Internet-based treatment, Tele therapy, Blended treatment, Therapeutic process, Working alliance, Depression, Covid-19

## Abstract

The current COV-19 pandemic increases the need for remote treatment. Among several provision strategies, tele groups have been tested as an efficient option. Still, the number of studies is comparably low, with a clear lack of studies investigating supposed treatment mechanisms.

Sixty-one mildly to moderately depressed participants from Salzburg, Bavaria, and Upper Austria were randomized to the intervention or a waiting list control group (RCT). The seven-week treatment comprised preparatory online modules, followed by personalized feedback and a subsequent tele group session.

Large treatment effects were observed for depression (CES-D: *d* = 0.99, *p* < .001; PHQ-9: *d* = 0.87, *p* = .002), together with large effects for cognitive behavioral skills (cognitive style, and behavioral activation, *d* = 0.88–0.97). Changes in skills mediated treatment outcomes for CES-D and PHQ-9, suggesting comparable mechanisms as in face-to-face therapy. Two typical moderators, therapeutic alliance, and group cohesion, however, failed to predict outcome (*p* = .289), or only exhibited statistical tendencies (*p* = .049 to .071). Client satisfaction, system usability, and treatment adherence were high.

Blending Internet-based and tele group interventions offers additional options for low-threshold care that is less dependent on population density, commuting distances, or constraints due to the current COV-19 crisis. Results indicate that the blended intervention is clinically effective by fostering core CBT skills. While findings suggest the notion that working alliance and group cohesion can be established online, their relevancy for outcomes of blended treatment needs to be further investigated.

## Highlights

• This study merged tele group therapy with an Internet intervention for the treatment of major depression.
• Standardized measures of service satisfaction and system usability indicated high treatment satisfaction.
• Investigated treatment mediators of the main outcomes supported the relevancy of cognitive and behavioural skill acquisition.
• While therapeutic alliance and group cohesion could be established, their relation to therapy outcome needs to be further tested.
• As a limiting factor sample size was relatively low which restricted process analyses to comparably simple path models.

## 1 Introduction

Depression is a common mental health disorder which imposes suffering, disability, and high economical costs on individuals, health systems, and society as a whole (Vigo et al., 2016; Welch et al., 2009). While a lot has been achieved in the development of effective treatments, open issues remain and further challenges need to be addressed in the striving for optimal treatment. Besides identifying causes and mechanisms of psychopathology, current psychiatric research prioritizes the implementation of early interventions, the expansion of access to treatment, as well as continuing research on eHealth. For example, the ROAMER consortium surveyed more than thousand mental health stakeholders in Europe and emphasized, among other priorities, on research on new technological approaches and their delivery (Wykes et al., 2015). More specifically, research should focus on real-time feedback for treatment adaption, as well as on the acceptability, long term effects, and cost-effectiveness of Internet interventions. More recent recommendations by expert groups support and expand these priorities (Bhugra et al., 2017; Holmes et al., 2018).

In the context of innovative depression treatment, several strategies can be pursued. Over the past two decades a steep increase in research on Internet interventions has been achieved, suggesting acceptable, efficient, and effective treatment can be realized by guided Internet interventions (Andersson, 2018), which also hold promise for depression prevention (Paganini et al., 2018; Sander et al., 2016). Simultaneously, a considerable number of studies investigated tele based treatments (Banbury et al., 2018; Berryhill et al., 2019; Gentry et al., 2019). Since outbreak of COV-19 in the beginning of 2020 the development and intense testing of digital interventions, especially tele therapy, has clearly speeded up due to national health care needs (Wind et al., 2020; Holmes et al., 2020). A recent review on videoconferencing for psychological depression treatment identified 33 articles of heterogeneous quality, including fourteen randomized controlled trials (Berryhill et al., 2019). Overall, findings suggest that depression can be successfully treated via tele interventions, and that comparable effects to in-person therapy can be expected.

Both strategies, Internet interventions and tele care, differ in important aspects of treatment application, and, thus, probably attract different patient groups. Most Internet interventions operate as structured digital interventions, providing core cognitive behavioral (CBT) techniques to patients that work autonomously or with asynchronous therapist guidance through a determined number of browser- or app-based treatment elements. At this, therapist guidance and therapeutic alliance have been shown to constitute important treatment facilitators, leading to improved outcomes (Baumeister et al., 2014; Flückiger et al., 2018). Tele treatment, on the other hand, typically translates personal treatment provision to remotely operating video sessions. This means, therapist contact is not restricted in time, and some rationales entail up to 25 or more synchronous video consultations (Banbury et al., 2018; Berryhill et al., 2019; Gentry et al., 2019). Simultaneously, treatment may be supported by worksheets, workbooks, or exercises, but many high-quality studies explicitly state that those materials were kept similar to original rationales (cf. Choi et al., 2014; Egede et al., 2015; Nelson et al., 2003), which, for example, limits the options for providing remote feedback on accomplished tasks, or for encouraging and affirming (cf. Mol et al., 2018).

Constituting a third treatment strategy, blended therapy (BT) combines personal sessions with browser- or app-based treatment elements. In a first review of 44 heterogeneous studies, Erbe and colleagues found high acceptability of BT (Erbe et al., 2017). Besides evidence from several high-quality trials indicating non-inferiority of time reduced treatment compared to full face-to-face therapy (Thase et al., 2018), first studies also suggest that face-to-face treatment augmented with digital interventions can lead to improved treatment outcomes (Berger et al., 2018; Zwerenz et al., 2017; Schuster et al., 2020a). On average, gains in effectiveness (*d* = 0.4) were comparable to combined antidepressant and psychotherapy treatment (Cuijpers et al., 2014; Hedge's *g* = 0.43). Importantly, BT has been reported to yield higher acceptance rates among addressed stakeholders compared to pure Internet interventions (Apolinário-Hagen et al., 2017; Dorow et al., 2018; Topooco et al., 2017; Schuster et al., 2020a). On the other hand, BT is geographically bound which represents an important limitation compared to Internet- and tele based interventions. This is why several studies have suggested to merge BT with tele therapy. For example, in the context of the Master Mind project (Vis et al., 2018) Etzelmueller et al. (2018) found high acceptability of blending video- and Internet-based individual therapy for depression in routine care. Patients were reported to value the provision of CBT content via the Internet-based treatment, while video-sessions assured the personal character of the service (Etzelmueller et al., 2018).

Regarding psychological group treatment, several studies investigated the merits of tele group therapy (tGT) and blended group therapy (bGT). A recent review on 17 tele health group interventions found high acceptability of the novel format, with similar outcomes for those comparing face-to-face and videoconference groups (Banbury et al., 2018). Qualitative studies unveiled perceived treatment factors, such as development of health knowledge, insights and skills. Furthermore, tGT was valued for the opportunity to engage with others and for the improved accessibility to psychological group interventions. Accordingly, high levels of therapeutic alliance and group cohesion were reported. These findings are being supported by two further recently published reviews (Berryhill et al., 2019; Gentry et al., 2019). One exception, however, were lower values in therapeutic alliance for tGT compared to face-to-face formats (Gentry et al., 2019). Although there are ongoing studies of therapy mechanisms in blended settings (Garety et al., 2021; Vernmark et al., 2019), data are sparse, which is a relevant limitation. Further studies are needed, especially accounts for treatment mediator studies.

As for bGT, only a small number of heterogeneous studies exist to date. bGT has at least been tested for anxiety disorders (Newman et al., 2014; Przeworski and Newman, 2004), substance use (Schuman et al., 2015), hoarding (Ivanov et al., 2018), and depression (Aguilera et al., 2017), with most interventions exhibiting a comparably short treatment duration of less than ten weeks. As a general finding, perceived acceptability of bGT was high but more evidence form large trials is needed. Regarding the therapeutic process, bGT was reported to provide new intervention elements, such as individual video exposure consultations during group based hoarding treatment (Ivanov et al., 2018). Other studies found ongoing evidence for reduced treatment duration for the blended format (Newman et al., 2014), or increased treatment effects, for example for substance use disorders (Carroll et al., 2014).

Regarding depression treatment, our workgroup developed several intense bGT groups focusing on psychoeducation and skill building for sub-clinical to moderate levels of major depression. A resource-oriented format was developed for early intervention (Schuster et al., 2017; Schuster et al., 2018a). Perceived acceptability of bGT in these pilot studies was high as 96% of participants agreed that technology could help to improve psychological group trainings, and 69% perceived technology as therapeutic factor (Schuster et al., 2018a). Findings are supported by another pilot study investigating bGT for Acceptance and Commitment-Therapy (Schuster et al., 2019). In a qualitative analysis of both interventions (Schuster et al., 2018b), patients valued the option of personal exchange, the shifts between treatment modalities, and the mobile app as a transfer facilitating element.

Apart from these promising findings, routine outpatient group treatment in rural areas or smaller towns can be difficult to install, hampering the implementation of those policies that aim at extending the provision of psychological treatment groups. Additionally, the limitations of the current Covid 19 crisis are aggravating the situation. We therefore tested whether the face-to-face group meetings in bGT can successfully be supplemented by tele sessions. More explicitly, we expected that tele bGT would constitute a highly acceptable treatment option, determined by low dropout rates together with high ratings of client service satisfaction and high perceived system usability for tele sessions and Internet-based elements. Furthermore, we assumed that seven weeks of intense tele bGT can result in high treatment effects on mild to moderate levels of major depression. Finally, we hypothesized that increases in CBT skills, such as behavioral activation and changes in cognitive style, would mediate the treatment effect, and that typical moderators of face-to-face groups, such as group cohesion and therapeutic alliance, would also be relevant during tele bGT.

## 2 Methods

### 2.1 Procedure

This study was approved by the local ethics committee (Ethical Review Board, University of Salzburg, Ref: EK-GZ 34/2018) and preregistered at the German clinical trial register (DRKS-ID: DRKS00017258). There are two groups - an experimental group and a waiting list control group (treatment commenced after termination of the experimental group) - and four repeated measurements (2 × 4 design), including one additional midterm assessment for group coherence and working alliance. Recruitment took place June to September 2019, with 6-month follow-up assessments terminating in June 2020. Eligible participants were selected on basis of the screening results and contacted by phone. Informed consent was obtained by five independent interviewers. Psychopathology was assessed by the structured interview Mini-DIPS (Margraf, 2013), which is the short version of the German DIPS (Diagnostic Interview for Psychological Disorders; Margraf et al., 2013). The Mini-DIPS assesses symptoms according to ICD-10. Prior to treatment, and DSM-5. Prior to treatment, eligible participants were required to complete the pre-assessment online questionnaire. Post-assessment took place one week after treatment termination.

### 2.2 Participant recruitment and selection

**Fig. 1:**
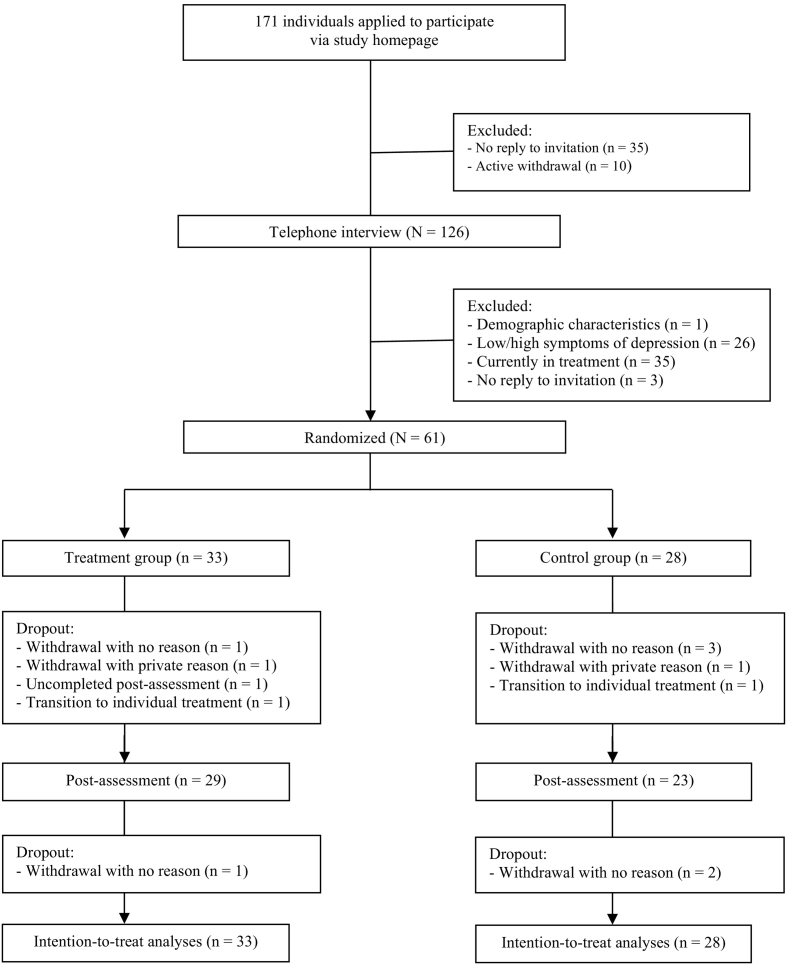
Study's flow chart.

The study was advertised online (e.g., www.depression.at) and in a local newspaper. All those interested were invited to fill out an online screening assessment, which was provided via the study homepage (www.ifd-salzburg.at). Recruitment ended after sufficient participants had been acquired. Randomization was based on a free random sequence generator (www.random.org), and concealed treatment allocation was implemented by an independent faculty member. Five psychologists with clinical experience conducted the telephone-based diagnostic interviews. Participants familiar with using personal computers and possessing Internet access, suffering from mild to moderate levels of major depression (CES-D cut-off >17 scale points), and in an age range from 18 to 65 were eligible. A small proportion of participants suffering from anxiety-related disorders were admitted due to comorbid major depressive disorder. Participants were excluded if they suffered from severe major depression, severe anxiety disorder, bipolar disorder, schizoaffective disorder, severe psychiatric and psychotic conditions, substance abuse, or suicidal ideation. Participants had to be excluded if they were in psychotherapy up to three months prior to the study period. Psychiatric medication was tolerated but had to be kept constant for the same duration in time. Additional information on participant recruitment can be gained from the CONSORT flow chart depicted in Fig. 1.

### 2.3 Measures

#### 2.3.1 Primary outcomes

The primary outcome, reduction of symptoms of depression, was measured by the German translation of the Center for Epidemiological Studies-Depression scale (CES-D, short version; Hautzinger and Bailer, 1993). This self-report questionnaire assesses depression-related emotions and motor function, as well as interactive, cognitive and somatic symptoms during the last two weeks with 16-items (4-step Likert-scale). The German version's cut-off value (CES-D > 17) was reported to have high discriminative validity (Hautzinger and Bailer, 1993), and its reliability has been shown to be excellent (Hautzinger et al., 2012). Test-retest reliability for CES-D was reported to be *r* = 0.59 (Radloff, 1977), which has been confirmed by more recent studies. Cronbach's alpha in the present study was.80.

The German version of the Patient Health Questionnaire-9 (PHQ-D; Löwe et al., 2002) was applied as a second measure for symptoms of depression. This valid and reliable self-rating questionnaire assesses symptoms of depression on 9 items, resulting in a sum score of 0 to 27. Cut-off values for mild, moderate, moderate to severe, and severe depression are >5, 10, 15, and 20. PHQ-9 exhibited a test-retest reliability of *ICC* = 0.81 (Löwe et al., 2004). Internal consistency of PHQ-9 in the present study can be classified as acceptable (Cronbach's alpha = 0.75).

#### 2.3.2 Secondary outcome measures

The very short form of the German Cognitive Style Questionnaire (CSQ-VSF-D; Huys et al., 2016) was applied pre- and post-treatment to assess changes in cognitive functioning. CSQ-VSF-D features the subscales negative consequences, implications, stability, and globality. Constituting the very short form of CSQ, the self-report measure still includes 27 items which relate to six text-based scenarios. Internal consistency of CSQ-VSF-D in the present study was excellent (Cronbach's alpha = 0.93).

Changes in depressive behavior were assessed by the German Behavioral Activation for Depression Scale (BADS; Teismann et al., 2016). This 25 items self-report measure consists of the four subscales activation, avoidance/rumination, work/school impairment, and social rated each on a 4-steps Likert-Scale. Internal consistency of BADS for the present study was good (Cronbach's alpha = 0.86).

#### 2.3.3 Assessment of treatment moderators

Patient-rated levels of the working alliance were assessed mid-treatment by the German Working Alliance Inventory – Short Revised form (WAI-SR; Wilmers et al., 2008). WAI-SR features the three dimensions task, bond, and goal, which are assessed by 3 × 4 items with a 5-step Likert-scale. Internal consistency of WAI-SR for the present study was high (Cronbach's alpha = 0.88). Normative data for the interpretation of the German WAI-SR are currently not available.

Additionally, group-related therapeutic alliance and group cohesion were assessed by the German Group Questionnaire (GQ-D; Bormann et al., 2011), a self-rating instrument featuring 30 items to be rated on a 7-point Likert-scale. The three subscales for group-related alliance are positive bonding relationship, positive working relationship, and negative relationship. Internal consistency of GQ for the present study was excellent (Cronbach's alpha = 0.94).

#### 2.3.4 Treatment satisfaction and system usability

Usability of the online intervention iFightDepression and the videoconference software zoom was assessed by the System Usability Scale (SUS; Brooke, 1996). The SUS is a self-rating scale for assessing quality of user interfaces. Empirical cut-offs of the 10-item 5-point Likert scale are graded from SUS > 85.5 (excellent usability), to SUS >71.4 (good usability), and SUS > 50.9 (acceptable usability) (Bangor et al., 2009). Cronbach's alpha for the current study can be classified as high (Cronbach's alpha = 0.86).

Participants' overall satisfaction with tele treatment was assessed by the German Client Satisfaction Questionnaire (CSQ-8; Schmidt et al., 1989; cut-off >24 indicates “good” satisfaction; Larsen et al., 1979) which is validated for Internet interventions as well (Boß et al., 2016). This widely used questionnaire addresses several aspects of service satisfaction and is based on an 8-item 4-point Likert scale. In this study Cronbach's alpha was excellent (Cronbach's alpha = 0.94).

#### 2.3.5 Intervention

The applied Internet intervention is focused on the reduction of depressive symptoms by increasing personal (antidepressant) skills. The applied treatment was developed by the European Alliance Against Depression (EAAD; cf. Justicia et al., 2017) and included seven Internet-based CBT sessions (www.ifightdepression.com). The browser-based program was originally designed to be guided by general practitioners (GPs), which means that weekly feedback on therapy progress was not foreseen in the original version. In the present study individual feedback on accomplished tasks was provided within two days after module completion (modules have been unlocked according to the course of intervention, so every week one model was assigned). Besides depression-related modules, three additional modules covering general health and social skills are implemented. Intervention details are provided in Box 1.

Box 1 **Overview of intervention elements.**

**Table:** 

Week	Internet intervention	Online feedback on tasks	Tele group session	Additional material during session
1			Opening, icebreaker session, introduction to treatment	
2	Relation of thoughts, feelings, and behavior Behavioral observation	✓	Welcome - reflection on content of Internet intervention – progress with exercises – open issues – practical support with frequent obstacles – additional content and exercises –feedback round and session closing	Exercise on values
3	Sleep and depression Sleep diary	✓		Worrying Sleep hygiene
4	Behavioral activation, activity scheduling	✓		Habits Activity list
5	Behavioral activation/self-management	✓		
6	Recognizing negative thoughts	✓		Acceptance exercise
7	Challenging negative thoughts	✓	Transfer and conclusion of the group

Alt-text: Box 1

Online modules alternated with weekly tele group conferences (multiple split screen view) delivered via zoom (www.zoom.us). According to general guideline recommendations for group treatment, tele sessions were provided in a double trainer setting. For the present study, four students (NS, SS, NN, and CJ) in their final year of clinical psychology formation (M.Sc.-level) delivered treatment. All student therapists had prior experience with conducting group therapy in clinical face-to-face settings, and possessed at least 480 h practical experience in psychiatry. Furthermore, the European Alliance Against Depression offers a structured training package to assure treatment fidelity and safety. All therapists underwent this training, and individual test calls were conducted to assure smooth start of tele group sessions. Sessions took place in a specially equipped room at University of Salzburg psychotherapy outpatient center. During the 90-min tele group sessions, each week's online content was consolidated, and open questions were discussed. The entire intervention content remained available to participants after treatment ended.

### 2.4 Statistical analyses

Statistical analyses were carried out using SPSS 24. Before starting main analysis, relevant requirements for parametric statistics were checked (e.g., scale distributions, homoscedasticity). Linear mixed models (LMM), with restricted maximum likelihood estimation (REML) and compound symmetry as covariance type, were used for estimating treatment effects. Missing values on outcome measures were analyzed according to the intention to treat principle (ITT), and baseline symptoms of depression were entered as covariate. The interpretation of all applied post hoc contrasts to identify significant differences between measurements was corrected for alpha-inflation (Bonferroni correction) based on number of outcomes. Putative mediators and moderators of treatment were investigated by the process macro, a simple and frequently used SPSS add-on (Hayes, 2017). Separate models were calculated for each outcome variable (CES-D and PHQ-9). Within- and between-group effects were calculated with pooled standard deviation and reported in Cohen's *d* (Cohen, 1988). G*Power software (Faul et al., 2007) was used to determine required sample size, resulting in an estimated sample size of *N* = 62, for a between-group effect of *d* = 0.8 (alpha-error α = 0.01, power β = 0.90, correlation among repeated measures = 0.3). For conservative estimates, we specified between effects of repeated analysis of variances as model specification. Parameter estimators were based on several previous studies from our group together with reviews for tele group treatment. As for applied moderation and mediation analyses, these would have required larger sample sizes, which is why the related findings need to be interpreted with awareness of limited statistical power. To test stability of mediation of the primary outcome CES-D, mediation analysis was also carried out for PHQ-9. In addition to statistical inference, we calculated reliable change (RCI) on basis of symptom change together with test-retest reliability of PHQ and CES-D. For CES-D patients had to experience change in symptoms of depression of 9.47. The respective value for PHQ-9 was 5.21 scale points.

## 3 Results

The 61 participants included 34 women (55.7%) and 27 men (44.3%) aged between 19 and 74 years (M = 38.95, SD = 12.07 years). Educational level was high, as almost half of the subjects (49.2%) owned a university or technical college degree. In addition, most participants were native German speaking and all participants reported routine Internet use. Regarding depressive disorder, the majority of participants suffered from recurrent MDD with a current moderate episode, followed by a recurrent MDD with a current mild episode. 21 subjects reported mostly anxiety-related comorbidities (34.4%). A proportion of 11.5% of the participants had an anxiety-related primary diagnosis but was included due to relevant depressive comorbidity. During treatment, we observed a low dropout rate of 14.75% (this compares to dropout rates of 34.2% for telehealth and 26.2% for CBT, Fernandez et al., 2015), and high presence of participants in the tele sessions (average = 81%). There were no significant differences between randomized groups at pre-assessment (all *p* > .05; all χ^2^ > 0.05). Additional information on participants' flow through the study, as well as on demographic and clinical characteristics, are provided in Table 1 and the CONSORT flow chart depicted in Fig. 1.

**Table 1:** Demographic and clinical participant characteristics.

Characteristic	*n*/*M*	*SD*/%
Age (*SD*)	38,9	(12.07)
Gender, female (%)	34	(55.7)
Education		
- Basic (%)	8	(13.1)
- High school degree/A-level (%)	23	(37.7)
- Academic	30	(49.2)
Employment		
- Fulltime	25	(41.0)
- Part time	11	(18.0)
- Student	11	(18.0)
- Unemployed	8	(13.1)
- Retired	4	(6.6)
- Else	2	(3.3)
Native German language	58	(95.1)
Daily Internet access	61	(100)
Primary diagnosis		
F32.0 Mild depressive episode	4	(6.6)
F32.1 Moderate depressive episode	5	(8.2)
F33.0 Recurrent depressive disorder, mild	13	(21.3)
F33.1 Recurrent depressive disorder, moderate	26	(42.6)
F33.4 Recurrent depressive disorder, currently remitted	3	(4.9)
F34.1 Dysthymia	3	(4.9)
F40.00/.01 Agoraphobia	2	(3.3)
F40.1 Social phobia	1	(1.6)
F41.0 Panic disorder	1	(1.6)
F42.2 Mixed obsessional thoughts and acts	1	(1.6)
F43.2 Adjustment disorder	2	(3.3)
Comorbidities	21	(34.4)

Note. *SD* = standard deviation.

### 3.1 Treatment effect on depression

According to the primary study aim, reduction of symptoms of depression was analyzed by means of separate linear mixed regression models (LMM) for CES-D and PHQ-9. Results indicated a significant interaction between group and time for CES-D *F*(1, 57.81) = 5.81, *p* = .019. At post-treatment, post hoc tests indicated significantly lower symptoms of depression in the experimental group compared to the control group (*p* < .001). For PHQ-9, the resulting pattern was similar. A significant interaction between group and time was found, *F*(1, 56.94) = 6.09, *p* = .017, and post hoc tests indicated significantly lower symptoms of depression in the experimental group (*p* = .002). Furthermore, there were no statistically significant changes from post-assessment to 6-month follow-up (all *p*'s > 0.05). Detailed information on means, standard deviations, and effect sizes is depicted in Table 2. Rates of reliable change varied as a function of assessment point. From screening to post assessment RCI was 55% for CES-D, and 52% for PHQ-9, with comparable proportions during 6-month follow-up (59% for CES-D, and 55% for PHQ-9). Proportions for pre- to post-assessment, however, where lower (38% for CES-D, and 31% for PHQ-9). Both questionnaires indicated one (3%) reliable deterioration in the experimental group.

**Table 2:** Means, standard deviations, and effect sizes for primary and secondary outcomes, as well as for putative treatment mediators.

		Screening		Pre-assessment		Post-assessment		Follow-up	Pre-post within	Post between	Pre-follow-up within
	*n*	*EM* (*SD*)	*n*	*EM* (*SD*)	*n*	*EM* (*SD*)	*n*	*EM* (*SD*)	*d* (*CI*)	*d* (*CI*)	*d* (*CI*)
CES-D											
EG	33	25.50 (5.21)	33	21.39 (5.51)	29	14.50 (5.49)	28	13.04 (6.85)	1.25 (0.66, 1.85)	0.99 (0.42, 1.57)	1.51 (0.79, 1.90)
CG	28	23.96 (5.60)	28	22.49 (5.50)	23	19.95 (5.46)	21	–	0.46 (−1.02, 0.09)		
PHQ-9											
EG	33	12.15 (4.17)	33	10.45 (3.04)	29	7.37 (3.02)	28	7.04 (5.51)	1.02 (−1.59, −0.44)	0.87 (0.30, 1.44)	1.12 (0.25, −1.29)
CG	28	12.57 (4.24)	28	10.76 (3.02)	23	10.01 (3.03)	21	–	0.25 (−0.80, 0.30)		
CSQ											
EG			33	81.97 (17.34)	29	63.33 (16.91)			1.09 (0.56, 1.62)	0.97 (0.38, 1.53)	
CG			28	80.32 (17.36)	23	79.61 (17.21)			0.04 (−0.59, 0.51)		
BADS											
EG			33	74.06 (20.68)	29	91.56 (20.46)			0.85 (0.33, 1.37)	0.88 (0.31, 1.45)	
CG			28	71.43 (20.70)	23	73.65 (20.38)			0.11 (−0.44, 0.66)		

Note: EG = experimental group; CG = control group; *EM* = estimated mean; *SD* = standard deviation; *d* = Cohen's *d* (based on the estimated mean); *p* = *p*-value; *CI* = 95% confidence interval. Follow-up data for CG missing due to transition to EG.

### 3.2 Treatment effect on cognitive and behavioral skills

Besides reduction of depressive symptoms, the study tested if combined Internet- and tele based treatment would result in increased cognitive and behavioral skills assessed by changes in cognitive style (CSQ-VSF-D) and in the level of behavioral activation (BADS). For changes in cognitive style LMM indicated a clear time by group interaction, *F*(1, 54.02) = 19.07, *p* < .001, with significant increases for the experimental group (*p* < .001). For changes in behavioral activation a comparable pattern emerged, with a significant group by time interaction *F*(1, 54.06) = 6.24, *p* = .016, and significant increases for the experimental group (*p* < .001). Again, detailed information on means, standard deviations, and effect sizes is provided in Table 2.

### 3.3 Mediators of treatment outcome

**Fig. 2:**
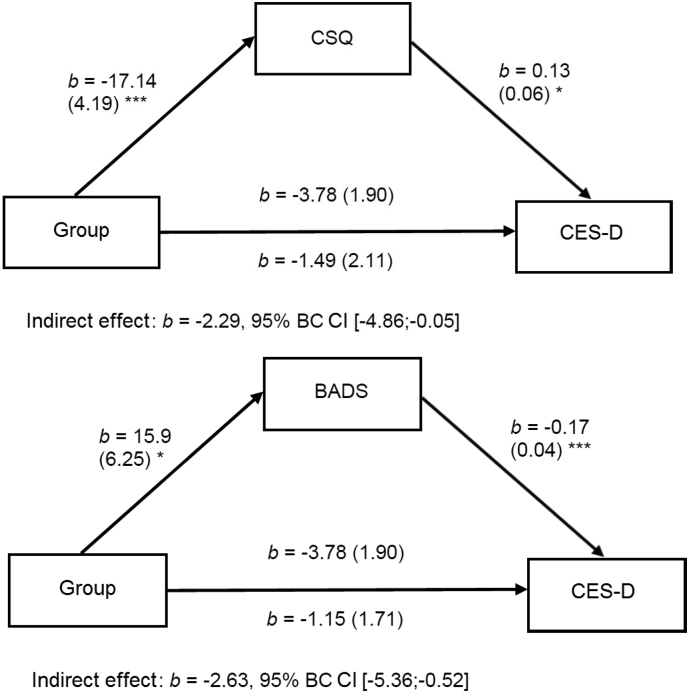
Putative mediators of treatment effect. Note. Outcomes for PHQ-9 are provided in Appendix 1. CSQ = Cognitive Style Questionnaire (CSQ-VSF-D); BADS = Behavioral Activation for Depression Scale; CES-D = Center for Epidemiological Studies-Depression scale. Note. Outcomes for PHQ-9 are provided in Appendix 1. CSQ = Cognitive Style Questionnaire (CSQ-VSF-D); BADS = Behavioral Activation for Depression Scale; CES-D = Center for Epidemiological Studies-Depression scale.

To investigate mechanisms of change, separate mediation analyses were calculated for CES-D and PHQ-9, with the latter results being provided in Appendix 1. Putative mediators of the CBT-based intervention were changes in cognitive style and behavioral activation, which were assessed pre- and post-treatment. Results are presented in Fig. 2, providing the unstandardized coefficients of single paths, together with bootstrapped 95% confidence intervals (BC CI's) for the indirect effect. Both mediators resulted in significant mediation effects for both depression outcomes (CES-D and PHQ-9), indicated by significant relations on paths A and B together with a 95% BC CI that did not include zero for the indirect effect.

### 3.4 Moderators of treatment outcome

Besides mediation of outcomes by cognitive and behavioral skills, perceived working alliance as well as group cohesion were assumed to moderate treatment success. Working alliance was assessed by the German version of the Working Alliance Inventory (WAI-SR). The total scale mean for the present study was *M* = 46.14 (*SD* = 7.42; BA 95% CI: 43.34, 48.68). As the German version does not provide normative data, ratings can be classified on basis of the Likert-scale labels. At this level, the average rating of *M* = 3.85 on a 5-point Likert-scale can be classified as quite high (representing 77% of the scale maximum). In addition, working alliance was assessed by the group‑leader subscale of the Group Questionnaire (GQ). Here, scale average was *M* = 68.39 (*SD* = 7.07; BA 95% CI: 65.46, 71.04), which represents 89% of the scale maximum. Finally, group cohesion was assessed by the group-member subscale of GQ. The corresponding scale average was *M* = 47.93 (*SD* = 7.70; BA 95% CI: 44.67, 50.52), which translates into 86% of the scale maximum. For each putative moderator separate models were calculated with baseline depression entering as covariate. Results of all three calculated models are summarized in Table 3. Five out of six moderator outcome relations indicated statistical tendencies or were just within the statistically significant range, and one relation was not statistically significant. The results from the WAI-SR and the GQ model indicated that a higher rating of working alliance predicted steeper reductions of depressive symptoms. In contrast, higher group coherence did not reduce or had only a tendency to reduce depressive symptoms.

**Table 3:** Putative moderators of treatment success.

Moderator	CES-D				PHQ-9			
	*b*	SE *b*	*t*	*p*	*b*	SE *b*	*t*	*p*
Model 1								
Constant	15.02	1.17	12.80	<0.001	7.33	0.57	12.77	<0.001
Working alliance (WAI-SR)	−0.32	0.16	−1.93	0.065†	−0.16	0.08	−2.07	0.049⁎
Baseline	0.38	0.21	1.83	0.079†	0.37	0.18	2.07	0.049⁎
Model 2								
Constant	14.75	1.14	12.92	<0.001	7.27	0.59	12.39	<0.001
Working alliance (GQ)	−0.33	0.16	−2.07	0.049⁎	−0.15	0.08	−1.89	0.071†
Baseline	0.26	0.21	1.29	0.208	0.31	0.19	1.64	0.113
Model 3								
Constant	14.75	1.19	12.36	<0.001	7.31	0.57	12.87	<0.001
Group coherence (GQ)	−0.18	0.17	−1.09	0.287	−0.15	0.07	2.02	0.054†
Baseline	0.36	0.21	1.72	0.097†	0.41	0.18	2.29	0.031⁎

Note. WA-SR = Working Alliance Inventory; GQ = Group questionnaire (member – leader, and member – group); CES-D = Center for Epidemiological Studies-Depression scale; PHQ-9 = Patient Health Questionnaire; *b* = regression coefficient; SE *b* = standard error of b.

⁎ *p* < .05.

† Statistical tendency (*p* < .1).

### 3.5 Treatment satisfaction and system usability

Participants' service satisfaction, as well as perceived usability of the tested Internet intervention and of the teleconference software was assessed by means of standardized questionnaires. Both scales were negatively skewed (*p* < .001 to *p* = .005). With a value of *M* = 27.61 (*SD* = 4.34; BA 95% CI: 25.94, 29.10) of 32 possible scale points, service satisfaction (assessed by the Client Satisfaction Questionnaire), was rated as “good” (cut-off >24; Larsen et al., 1979). Furthermore, system usability (SUS) of the iFightDepression intervention was *M* = 79.19 (*SD* = 12.78; BA 95% CI: 73.75, 84.29), which can be classified as “good” (cut-off >71.4; Bangor et al., 2009). Finally, usability of the teleconference software zoom was *M* = 85.54 (*SD* = 15.73; BA 95% CI: 79.53, 90.71), which indicates “good” usability, right on the margin to “excellent” usability.

## 4 Discussion

This study investigated the acceptability, effects, and mechanisms of tele bGT in the treatment of mild to moderate MDD. The novel format combines two digital formats that usually exist rather independent from each other, psychological tele treatment and Internet-based interventions. Seven week intensive treatment based on preparatory online modules, remote feedback, and subsequent tele group consultations resulted in large effects on mild to moderate symptoms of MDD. Furthermore, acquisition of core CBT skills (changes in cognitive style and behavioral activation) was found to meditate reduction of symptoms of depression, indicating comparable treatment mechanisms as known from face-to-face therapy. Additional mechanisms were investigated by moderation analysis, but these findings resulted in a less clear pattern. Finally, treatment acceptability was high, as indicated by low attrition rate together with high ratings of client service satisfaction and system usability.

Considering the general acceptability of tele bGT, high service satisfaction and system usability were reported by our participants. This appraisal is supported by reviews on tele group treatment (Banbury et al., 2018; Berryhill et al., 2019; Gentry et al., 2019), as well as by those studies conducted at our university outpatient psychotherapy center (cf. Schuster et al., 2018a, Schuster et al., 2018b, Schuster et al., 2018c; Schuster et al., 2019). At this, tele groups augmented with browser- and app-based features (e. g. diaries, informational videos) appear to be in an interesting spot on the mental health spectrum: they facilitate remote treatment, possess sufficient scalability, and maintain some form of personal live contact. Supporting evidence comes from a qualitative study investigating individual treatment (Etzelmueller et al., 2018). According to the authors, patients valued the Internet intervention for a deeper reflection of treatment principles, while tele sessions were reported to provide the personal character of the service. From the individual perspective, it appears plausible that different intervention formats attract different groups of patients. This suggests that high service satisfaction can be expected by individuals seeking this kind of treatment. In our previous studies these were individuals looking for personal exchange, or being interested in innovative or proactive treatment (Schuster et al., 2017; Schuster et al., 2018b). Suboptimal therapeutic processes appear more probable for individuals expecting talking therapies, for individuals being less enthusiastic about digital treatment, or exhibiting complex issues that better should be treated by means of individual therapy. In this regard, a recent study found a positive personal advantage index for patients that were retrospectively matched to face-to-face or blended treatment according to sociodemographic and clinical characteristics (including treatment expectancy) (Friedl et al., 2020). Regarding adverse treatment outcomes and therapy dropouts, our findings indicate low proportions of reliable deterioration (3%) and premature treatment termination (14.75%). The deterioration corresponds to rates known from Internet-based (5.8%; Rozental et al., 2017), as well as from face-to-face therapy (4%; Cuijpers et al., 2018).

Regarding the effects of combined treatment, findings are in line with recent reviews on tele group treatment (Banbury et al., 2018; Berryhill et al., 2019; Gentry et al., 2019) and blended therapy (Erbe et al., 2017), indicating that high treatment effects can be realized by both types of treatment. Importantly, the present effects were achieved within a comparably short time period of seven weeks, with each week featuring one preparatory online module (including asynchronous feedback) and one subsequent tele group session. Compared to two previous studies on face-to-face bGT that used the same or comparable outcome measures (Schuster et al., 2018a; Schuster et al., 2019), current pre-post effect sizes appear a bit smaller, but small sample sizes impede further inferences about meaningful differences. Simultaneously, ongoing evidence suggests that combined treatment may actually lead to augmented outcomes (Berger et al., 2018; Zwerenz et al., 2017; Schuster et al., 2020a).

As tele bGT was based on core CBT principles, we were interested in observable differences in dysfunctional cognitions (Cristea et al., 2015) and behavioral activation (Hopko et al., 2003). Statistical analysis revealed rather large effects for both secondary outcomes, a pattern that was found in previous bGT studies for depression (Schuster et al., 2017; Schuster et al., 2018a); even though on study found smaller effects on secondary outcomes (Schuster et al., 2019). Furthermore, these skills were related to treatment success, as indicated by significant mediation analyses for both assessed skills and both self-report measures of depression. Regarding the interpretation of proposed mechanisms of change, it is important to state that statistical mediation analysis can depict relations, but it cannot fully prove their assumed causality (cf. Kazdin, 2007; Lemmens et al., 2016).

Besides the impact of CBT skills on depression, we tested whether typical treatment moderators could be identified in tele bGT. For face-to-face groups, putative moderators typically include the therapeutic working alliance and cohesion among group members (cf. Burlingame et al., 2011; Holmes and Kivlighan, 2000; Yalom and Leszcz, 2005). From a mere descriptive point of view, we observed relatively high levels of perceived working alliance and group cohesion, suggesting that such processes can be realized in tele bGT as well. This interpretation is in line with current tele group studies (Banbury et al., 2018; Berryhill et al., 2019; Gentry et al., 2019; Simpson and Reid, 2014). If those process variables can actually be expected to be on-pars with face-to-face treatment, however, needs to be further established as some studies reported slightly lower levels (Greene et al., 2010). As for their relation with therapy outcome, a recent review found significant relations (of approximately *r* = 0.3) between therapeutic alliance and outcomes for both face-to-face and Internet interventions (Flückiger et al., 2018). Ongoing evidence from tele therapy and blended interventions is less conclusive, as first studies on the topic failed to identify such relations (Kooistra et al., 2020; Vernmark et al., 2019). Although we found some indications for alliance-outcome relations in the current sample, these were only supported by statistical trends. Thus, more studies are needed to draw conclusions on whether those factors are of equal relevancy for those novel formats.

This study has noteworthy strengths and limitations that need to be considered when interpreting its findings. Among its most important strengths, the pre-registered RCT investigated novel ways of digital treatment provision, which is a current research priority. Besides the acceptability of tele bGT, the study tested potential moderators and mediators of therapy success. According to recommendations, more than one mediator variable was assessed and tested for multiple outcomes (CES-D and PHQ-9). The resulting patterns converged to a wide extend, suggesting reliable mechanisms.

This study exhibits the following noteworthy limitations. Concerning assessment of symptoms of depression, this study was based on self-reports. Findings could differ, if observer-rated assessments were implemented. Future studies should include observer ratings to assure validity of findings. Furthermore, reported effect sizes are based on a passive control group study design, which is why smaller effects must be assumed if an active control group had been used. Evidence from preferably larger studies with active comparators or dismantling designs is needed to estimate the benefits of combining tele treatment with Internet interventions. Regarding mediation analysis, this study did not follow recent recommendations for minimal sample size in process studies (e.g., >40 per treatment arm for mediation analysis; cf. Lemmens et al., 2016). Besides the observed convergent patterns of therapy processes, the study therefore needs to be interpreted with awareness of limited sample size. Future process studies should implement intense assessment to provide more measurement points and to optimize statistical power (cf. Lemmens et al., 2016; Schuster et al., 2020b). A further limitation relates to study context as recruitment was reported to impact clinical characteristics in Internet-based treatment for depression (Lindner et al., 2015). Even though the study was conducted in an affiliated outpatient psychotherapy center, the included participants were self-recruited. While several studies suggest good generalizability in terms of effectiveness in routine care (Königbauer et al., 2017), it is also possible that recruitment form general population results in less pronounced effects (cf. Richards and Richardson, 2012) or less optimistic appraisals of service satisfaction or system usability. Simultaneously, other factors might act in the opposite direction. For example, student therapists unfamiliar with routine digital care applied the treatment. While they were experienced with conducting face-to-face groups, they lacked prior experience with tele treatment and Internet interventions. It could therefore be that more (digitally) experienced therapists acting in established workflows would actually produce better outcomes, as suggested by findings from routine anxiety treatment (El Alaoui et al., 2015).

## 5 Conclusions

The study adds to the ongoing evidence for tele group treatment. It combined an online intervention for depression, which had been primarily designed as a self-guided intervention, together with weekly tele group meetings. This format assures remote service provision while maintaining some form of personal contact (tele group meeting). Findings revealed high treatment effects in a comparably short time period but should be interpreted with respect to the small sample size and the early stage of research. Information on findings on intervention mechanisms largely support the assumption that mechanisms known from classic face to face therapy also apply in the tele setting. However, the moderating role of factor such as working alliance require further investigation, which currently is perpetuated by the COV-19 pandemic.

## Role of funding

No funding received.

## Declaration of competing interest

The authors declare no competing interests.

## Appendix Supplementary data

Appendix 1 Fig. 1. Results of mediation analysis for Patient Health Questionnaire (PHQ-9). Appendix 1
