# Supplementary figures and images for: Blending Internet-based and tele group treatment: Acceptability, effects, and mechanisms of change of cognitive behavioral treatment for depression
Source: Internet Interv. 2022 Jun 1;29:100551. doi: 10.1016/j.invent.2022.100551 (PMC9204733; doi:10.1016/j.invent.2022.100551)

Appendix 1


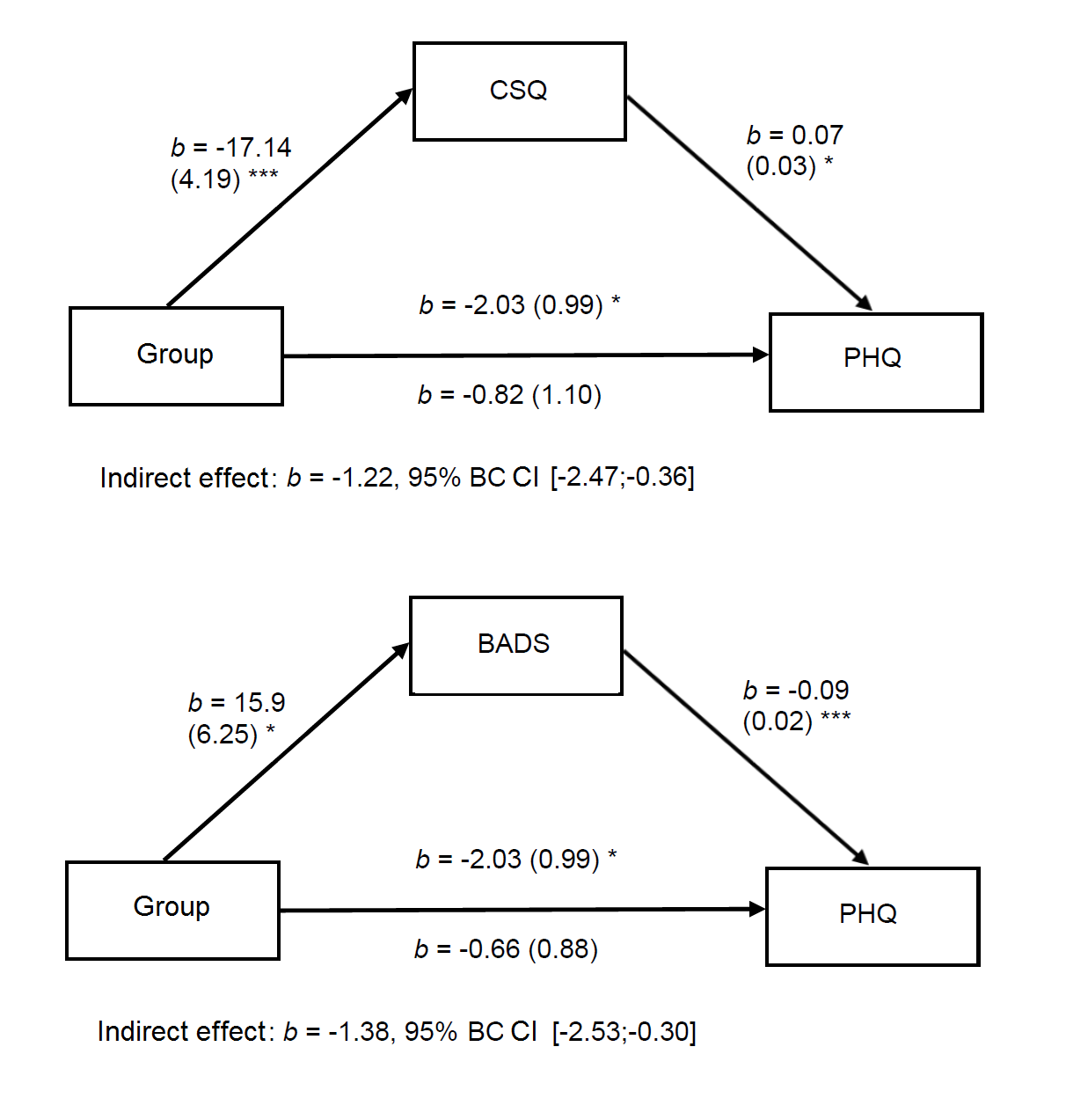


Figure 1. Results of mediation analysis for Patient Health Questionnaire (PHQ-9).

Supplement: Appendix 1 — Fig. 1. Results of mediation analysis for Patient Health Questionnaire (PHQ-9). [file mmc1.docx]
